# WRKY Transcription Factor Responses and Tolerance to Abiotic Stresses in Plants

**DOI:** 10.3390/ijms25136845

**Published:** 2024-06-21

**Authors:** Ziming Ma, Lanjuan Hu

**Affiliations:** 1 Jilin Provincial Engineering Laboratory of Plant Genetic Improvement, College of Plant Science, Jilin University, Changchun 130062, China; 2 Max-Planck-Institute of Molecular Plant Physiology, Am Muehlenberg 1, Golm, 14476 Potsdam, Germany; 3 Plant Genetics, TUM School of Life Sciences, Technical University of Munich (TUM), Emil Ramann Str. 4, 85354 Freising, Germany

**Keywords:** WRKY transcription factor, abiotic stress response and tolerance, target gene, plant growth and development

## Abstract

Plants are subjected to abiotic stresses throughout their developmental period. Abiotic stresses include drought, salt, heat, cold, heavy metals, nutritional elements, and oxidative stresses. Improving plant responses to various environmental stresses is critical for plant survival and perpetuation. WRKY transcription factors have special structures (WRKY structural domains), which enable the WRKY transcription factors to have different transcriptional regulatory functions. WRKY transcription factors can not only regulate abiotic stress responses and plant growth and development by regulating phytohormone signalling pathways but also promote or suppress the expression of downstream genes by binding to the W-box [TGACCA/TGACCT] in the promoters of their target genes. In addition, WRKY transcription factors not only interact with other families of transcription factors to regulate plant defence responses to abiotic stresses but also self-regulate by recognising and binding to W-boxes in their own target genes to regulate their defence responses to abiotic stresses. However, in recent years, research reviews on the regulatory roles of WRKY transcription factors in higher plants have been scarce and shallow. In this review, we focus on the structure and classification of WRKY transcription factors, as well as the identification of their downstream target genes and molecular mechanisms involved in the response to abiotic stresses, which can improve the tolerance ability of plants under abiotic stress, and we also look forward to their future research directions, with a view of providing theoretical support for the genetic improvement of crop abiotic stress tolerance.

## 1. Introduction

Crops are an indispensable and important resource in human life, carrying people’s survival and development. From planting to harvesting, from processing to consumption, food has accompanied the historical process of humankind and profoundly influenced the way of life and social structure of humankind [1]. Crops are able to provide humans with an abundance of carbohydrates, proteins, and multivitamins to maintain the normal physiological functions of the human body [2]. In conclusion, ensuring the normal development of crops is an important guarantee for the sustainable and stable development of human society [3].

Plants are subjected to a variety of stresses during growth and development, such as drought, temperature extremes, pests, diseases, etc., of which abiotic stresses are the major threats affecting the growth of food crops [4,5,6,7]. Currently, numerous studies have been conducted on the response of plants to abiotic stresses, for example, the response to cold temperature stress, drought stress, salt stress, heavy metal stress, etc. In order to survive, plants need to make a series of morphological and physiological-biochemical metabolic adjustments to adapt to adversity when subjected to abiotic stress [8,9,10]. First, when the outer cell membrane is stimulated by abiotic stress, second signalling molecules such as reactive oxygen species (ROS) are produced. The second signalling molecules then stimulate the intracellular membrane, which, by regulating intracellular Ca^2+^ levels, initiates a protein phosphorylation cascade reaction and produces phosphorylated protein molecules that are involved in the cytoprotection of proteins or transcription factors that regulate specific stress-regulated genes. The products of some of these genes can participate in the production of abscisic acid (ABA) and ethylene (ETH), which activates the expression of transcription factors. The transcription factors are able to bind specifically to their downstream target gene promoter sequence portions, thereby regulating the expression of downstream functional genes and ultimately improving the plant’s ability to cope with abiotic stresses [11,12]. Studies have been deepened to the level of molecular mechanisms, and WRKY transcription factors, which are one of the largest families of transcription factors in plants, have gained great attention. Nowadays, a large number of WRKY transcription factor genes have been identified, and they are widely involved in the regulation of plant secondary metabolism, abiotic stress, growth, and development [13,14,15]. Wang et al. found a WRKY transcription factor, ZmWRKY40, was induced by drought, high salt, high temperature, and ABA. *ZmWRKY40* overexpression increased drought tolerance in transgenic *Arabidopsis thaliana* due to the modulation of the expression of downstream abiotic stress genes and reduced the ROS content of the transgenic lines through the enhancement of peroxide dismutase (POD) and catalase (CAT) activities under drought stress [16]. Yin et al. found a WRKY transcription factor, PcWRKY33, specifically binds to the W-box in the promoters of downstream target genes to regulate their expression. The expression of *PcWRKY33* can be induced by various abiotic stresses. Overexpression of *PcWRKY33* reduced tolerance to salt stress in *Arabidopsis*. In transgenic plants, the expression of stress-related genes was decreased, the ability to maintain Na^+^/K^+^ homeostasis was weakened, the activity of ROS scavenging enzymes was decreased, and the accumulation of ROS was increased after salt stress [17]. Zhang et al. identified a WRKY transcription factor, OsWRKY63, negatively regulates cold tolerance in *Oryza sativa*. Overexpression of *OsWRKY63* lines was more sensitive to cold stress, and the knockout mutant line showed higher cold tolerance. OsWRKY63 was able to repress the expression of *OsWRKY76*, and the *OsWRKY76*-knockout mutant lines showed significantly lower cold tolerance and suppressed cold-induced expression of the five *OsDREB1* genes [18]. Ma et al. found that the tomato WRKY transcription factor SlWRKY57 acts as a negative regulator in the salt stress response by directly attenuating the transcription of salt-responsive genes (*SlRD29B* and *SlDREB2*) and an ion homeostasis gene (*SlSOS1*) [19]. Devaiah et al. found WRKY75 was located in the nucleus and was differentially induced in plants with phosphate (P_i_) deficiency. When WRKY75 expression was inhibited, the expression of several genes involved in the P_i_ starvation response decreased, and the lateral root length and number as well as the number of root hairs significantly increased [20]. Therefore, the important role of WRKY transcription factors in the regulation of abiotic stress and plant growth and development has made it become a popular gene family for plant stress breeding research. 

This paper reviews the structural domains and classification of WRKY transcription factors and which transcriptional regulation downstream genes are involved in the response to abiotic stress. It also discusses the research progress on the role of WRKY transcription factors in regulating the response of plants to abiotic stress, which will provide a theoretical reference for the future study of the ability of WRKY transcription factors to improve the ability of plants to cope with abiotic stresses.

### 1.1. Structural Domains and Classification of WRKY Transcription Factors

**Figure 1:**
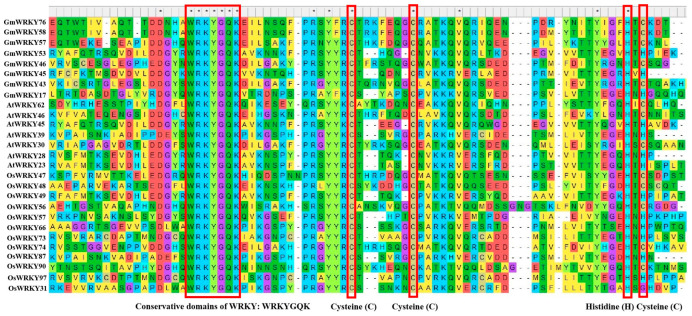
Conserved domains of WRKY family transcription factors in *Glycine max*, *Arabidopsis thaliana,* and *Oryza sativa*. These asterisks “*” and the red frames above the maps indicate the domains of conservative cysteine (C), cysteine (C), histidine (H), and cysteine (C), respectively.

**Figure 2:**
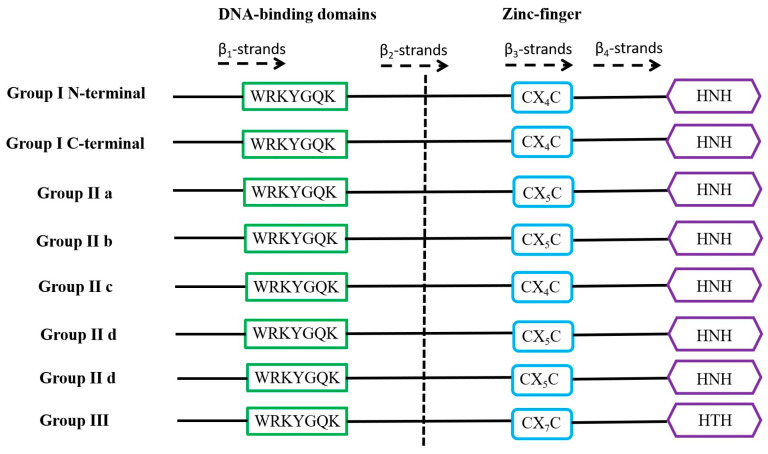
Domain structures of different WRKY subfamilies in higher plants. The WRKY motif, the cysteines, and the histidines that form the zinc finger are shown in boxes. The 4 β-strands are shown with dashed arrows.

WRKY is a family of transcription factors unique to plants and defined as WRKY transcription factors because their protein sequences contain a DNA-binding domain with the highly conserved amino acid sequence WRKYGQK at the N-terminus of the domain [21]. However, in a few WRKY proteins, there are different types of mutations in the WRKYGQK amino acid sequence, mainly WRKYGEK, WRKYGMK, WRKYGKK, WSKYEQK, or WIKYGEN (Figure 1). The WRKY transcription factor proteins are usually composed of 60 amino acids, and this sequence recognises the W-box [TGACCA/TGACCT] and some similar W-boxes containing the TGAC core structure in the homoeopathic element and specifically binds to them to regulate the downstream target genes [22,23]. The neighbouring sequences of the TGAC core structure determine the priority of the WRKY transcription factor binding sites [24]. A large number of previous studies have shown that most of the promoters of genes related to abiotic stresses contain more than one W-box sequence, which explains the ability of the WRKY family of transcription factors to be widely involved in the regulation of the expression of many plants’ abiotic stress-related genes [25] (Table 1). Typically, WRKY transcription factors contain at least one WRKY structural domain at the N-terminal end and an atypical zinc-finger structure at the C-terminal end [26]. Based on the number of WRKY-binding domains and the characteristics of the zinc finger-like motifs, they can be classified into three groups: Group I contains two WRKY-binding domains with C_2_-H_2_ motifs (C-X_4-5_-CX_22-23_-H-X_1_-H); Group II contains one WRKY-binding domain with C_2_-H_2_ motifs. Group II can be generally divided into five subgroups (II a, II b, II c, II d, and II e). Group III contains a WRKY-binding domain and a different zinc finger-like motif, C_2_-H-C (C-X_7_-C-X_23_-H-X_1_-C). Analyses based on phylogenetic data indicate that WRKY families in higher plants are more accurately classified into groups I, II a + II b, II c, II d + II e, and III [27,28] (Figure 2). 

By summarising the localisation, self-activating activity, and binding elements of published WRKY family transcription factors, we have found that most of the identified WRKY family transcription factors are localised in the nucleus, have self-activating activity, and are able to bind to W-box elements. (Refs. [46,47,48,49,50,51,52] are cited in Supplemental Table S1).

### 1.2. W-box cis-Element in the Promoter Region of WRKY Downstream Genes

It has been shown that WRKY transcription factors can specifically bind to DNA through the cis-acting element W-box (5′-[TGACCA/TGACCT]-3′) in the promoters of target genes to regulate the expression of related genes and affect plants under abiotic stresses, plant growth, and development. The core sequence of the W-box is “TGAC”, so the W-box can be used for the prediction of the target genes of WRKY transcription factors [21]. Different WRKY transcription factors bind to different sequences near the W-box, which affects the selectivity and strength of WRKY transcription factor binding [53]. Mutations in the conserved sequence WRKYGQK in the WRKY structural domain or changes in any nucleotide in the W-box reduce the binding activity of WRKY transcription factors to DNA, whereas the substitution of conserved cysteine (Cys) and histidine (His) residues in the C-terminal zinc-finger structure removes their DNA-binding activity [54].

## 2. Response and Tolerance of WRKY Transcription Factor Family to Abiotic Stresses in Plants

Plants may encounter the effects of a wide range of abiotic stresses during growth and development, including drought, temperature extremes (high temperature and low temperature), salinity, heavy metal stresses, and nutrient deficiencies. With climate change and increasing weather extremes, the impact of abiotic stresses on crop production is increasing, leading to growth retardation, quality deterioration, and yield reduction [55,56]. Under abiotic stress conditions, WRKY transcription factors activate or suppress the transcription of downstream genes to regulate the expression of abiotic stress-responsive genes or the direct regulation of abiotic stress-responsive gene expression, activate the defence mechanism against abiotic stresses in crops, and improve crops’ resilience to ensure grain yield under abiotic stresses [57,58].

### 2.1. Molecular Mechanisms of WRKY Transcription Factors Associated with Drought Stress

**Figure 3:**
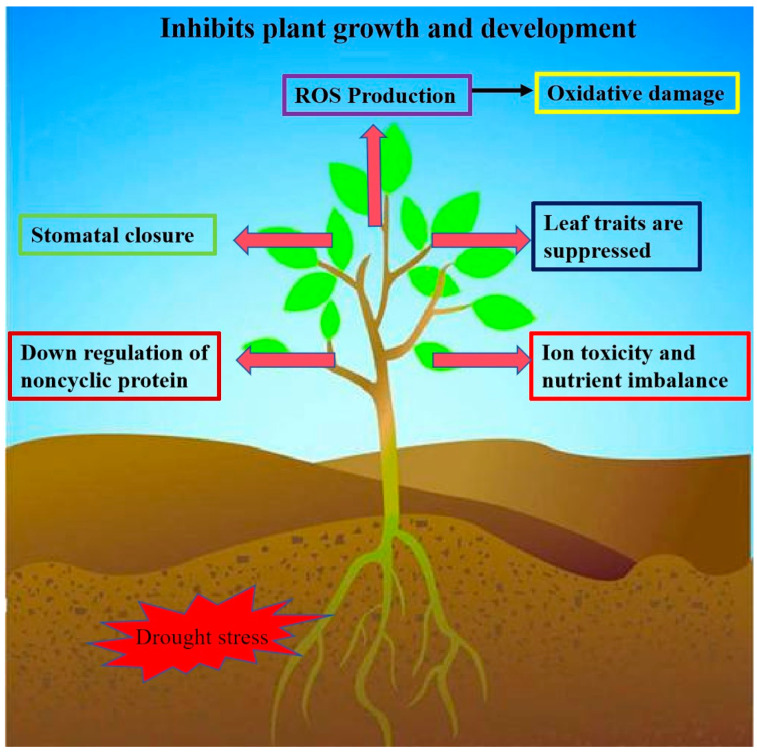
Impacts of drought stress on plants. Overexpression of WRKY transcription factor genes in plants can reduce ROS production, stomatal closure, and downregulation of noncyclic proteins, and leaf traits, ion toxicity, and nutrient imbalance are suppressed. This allows plants to improve their water retention capacity to cope with plant survival rate under drought stress.

Drought is one of the major environmental factors affecting plant growth and development and crop yield (Figure 3). Many WRKY transcription factor genes have been identified to regulate drought tolerance in plants, and overexpression lines or knockdown lines of these WRKY genes have improved drought tolerance and even seed yield in Arabidopsis thaliana, Nicotiana tabacum, Glycine max, Oryza sativa, Triticum, and cotton [55,56,57,58]. It has been shown that WRKYs can improve plants’ tolerance to drought stress by reducing their H_2_O_2_ content through the ROS-scavenging system. For example, overexpression of MdWRKY70L in Nicotiana tabacum reduced H_2_O_2_ and O_2_^−^ accumulation and enhanced drought tolerance in transgenic plants [59]. Overexpression of GmWRKY17 enhances drought tolerance in soybean, and it positively regulates drought tolerance in soybean by activating the expression of the drought-inducible gene GmDREB1D and the ABA-associated gene GmABA2 by combining their promoters [49]. The expression of *ZmWRKY106* was significantly induced by drought, high temperature, and exogenous ABA. *ZmWRKY106* overexpression lines in *Arabidopsis* showed increased tolerance to drought and high temperature. Under drought stress, ZmWRKY106 reduced the ROS content in the transgenic lines by enhancing superoxide dismutase (SOD), POD, and CAT activities [60]. Duan et al. found that the transcript abundance of MdWRKY56 was upregulated under drought stress. A MdWRKY56 overexpression line exhibited lower electrolyte leakage, malondialdehyde (MDA) content, ROS accumulation, proline content, and antioxidant enzyme activities [61]. Zhang et al. found that ChaWRKY40 may enhance hazelnuts’ drought tolerance by positively regulating the ChaP5CS gene’s expression to increase the proline content. In the wild type, the expression of ChaWRKY40 and ChaP5CS increased with the increase in the PEG-6000 concentration in the leaves and the gradual decrease in the relative water content in the leaves [62]. Wang et al. found that the WRKY transcription factor EjWRKY17 was identified in Eriobotrya japonicaloqua, which was significantly upregulated in leaves by melatonin treatment during drought stress. The EjWRKY17 overexpression line was able to increase drought tolerance in plants, which had low water loss, limited electrolyte leakage, and lower levels of ROS and MDA compared with the wild type [63]. Huang et al. found that MfWRKY40 promoted primordial root length elongation, increased water uptake, and reduced water loss under stress, and the antioxidant capacity of the overexpression lines was also significantly enhanced, as evidenced by the higher chlorophyll content and antioxidant enzyme activities and less malondialdehyde and ROS accumulation [64].

Drought is one of the major abiotic stresses that limit plant growth and development and reduce crop yield. Many members of the WRKY transcription factor family have been found to be able to respond to drought stress by binding downstream to the W-box element in the promoter region of drought stress-related genes and regulating the expression of these genes, which ultimately improves the ability of plants to cope with drought stress.

### 2.2. WRKY Transcription Factors Involved in Response to Temperature Stress

Temperature is considered the major abiotic stress for plants. Extreme high or low temperatures can lead to severe effects on plants, resulting in plant mortality and extensive agricultural economic losses. It is, therefore, important to increase the tolerance of plant cells to drastic changes in temperature and necessary to protect food production. WRKY transcription factors help plants resist temperature changes by regulating the expression of genes involved in temperature stress, and they also respond to extreme temperatures by regulating the expression of genes involved in the ABA response [65,66,67].

#### 2.2.1. WRKY Transcription Factors and High-Temperature Stress

When the temperature exceeds the maximum upper limit of the temperature to which a plant can adapt, it has an injurious effect on the plant and stunts its growth and development. High temperatures can also weaken photosynthesis and enhance respiration, causing plants to over-consume their own energy and causing them to die from long-term starvation. High temperatures can also disrupt the water balance of plants, leading to the accumulation of harmful metabolites in the body. Therefore, high temperature is a significant stress factor that limits the normal growth and development of plants. Nowadays, more and more researchers are focusing on high-temperature stress in their studies [68,69]. He et al. found that the WRKY transcription factors TaWRKY1 and TaWRKY33 were identified in *Triticum aestivum* L. *TaWRKY33* overexpression lines showed enhanced heat stress tolerance. *TaWRKY1* was slightly upregulated by high temperatures, and ABA was downregulated by low temperatures. TaWRKY33 was involved in the response to high and low temperatures. The overexpression of *TaWRKY1* and *TaWRKY33* activated several stress-related downstream genes and promoted root growth in *Arabidopsis* under various stresses [70]. Wang et al. identified a WRKY family transcription factor *SlWRKY3* that was induced and upregulated under heat stress, whereas a knockout strain of *wrky3* resulted in reduced heat stress tolerance. The overexpression of *SlWRKY3* accumulated less ROS, whereas *wrky3*-knockout lines accumulated more ROS under heat stress. They concluded that *SlWRKY3* activated the expression of a range of abiotic stress-responsive genes involved in ROS scavenging [71]. Wu et al. performed transcriptome analysis on lily (*Lilium longiflorum*) and identified the WRKY family transcription factor gene *LlWRKY22*, whose expression was activated at high temperatures, and the overexpression of *LlWRKY22* in *Lilium longiflorum* increased its heat tolerance and activated the expression of heat-related *LlDREB2B* genes, which play a positive role in the regulation of heat tolerance [72]. Balfagón et al. found that the WRKY family transcription factor *AtWRKY48* was able to negatively control *Arabidopsis* acclimation to a combination of high light and heat stress and that the *AtWRKY48* gene’s expression was reduced by jasmonic acid (JA) under these conditions [73]. 

#### 2.2.2. WRKY Transcription Factors and Low-Temperature Stress

Temperature is one of the main environmental factors affecting the normal growth of plants and is necessary to ensure the normal growth of plants. Too low a temperature can cause stress to plants, affecting the growth and development of the plants. With the change in the global climate, low temperature has become an agrometeorological disaster. When the temperature drops to the lowest limit that plants can tolerate, it causes crop growth obstacles and damage to the fruiting organs and ultimately leads to the inability for normal growth and fruiting, which results in a substantial reduction in crop yields. Therefore, the study of the molecular mechanisms of plants’ responses to low-temperature stress is of great scientific significance in improving the cold tolerance of crops [74,75]. Mi et al. identified a WRKY family transcription factor, CsWRKY21, in tea tree. *CsWRKY21* was induced by low temperatures and expressed six times more compared with the control [76]. Yu et al. identified 42 *PgWRKY* genes of seven subclasses in the genome of *Platycodon grandiflorus*. Among them, the expression of *PgWRKY26* significantly increased after 6 h under cold stress [77]. Wang et al. identified a cold-inducible WRKY gene, *PmWRKY57*, which was cloned in *P. mume*. A *PmWRKY57* overexpression line increased cold tolerance in *Arabidopsis*. Under cold treatment, the transgenic lines had significantly lower malondialdehyde contents and significantly higher superoxide dismutase activity, peroxidase activity, and proline contents in leaves than in wild-type plants. The expression levels of cold-responsive genes such as *AtCOR6.6*, *AtCOR47*, *AtKIN1*, and *AtRCI2A* were upregulated in transgenic *Arabidopsis thaliana* leaves compared with the wild type [78]. Liu et al. isolated an uncharacterised WRKY family transcription factor, VvWRKY28, in Beichun (*V. vinifera x V.amurensis*). Cold treatments can induce the high expression of *VvWRKY28. VvWRKY28* overexpression lines improved the tolerance to low temperatures in *Arabidopsis*. Among them, MDA contents decreased, chlorophyll and proline contents increased, and SOD, POD, and CAT activities increased in the *VvWRKY28* overexpression lines. In addition, WRKY28 may be associated with the regulation of the expression of downstream genes associated with cold stress (*RAB18*, *COR15A*, *ERD10*, *PIF4*, *COR47*, and *ICS1*) [79]. Wang et al. identified a new WRKY family transcription factor, SlWRKY50, in *Solanum lycopersicum.* SlWRKY50 responds to cold stimuli and plays a key role in JA biosynthesis. *SlWRKY50* overexpression lines increased cold resistance in tomato, leading to higher levels of Fv/Fm, antioxidative enzymes, allene oxide synthase expression, and JA accumulation [80].

### 2.3. WRKY Transcription Factors in Response to Salt Stress

When the salt concentration in the soil is too high, it leads to the dehydration of plant cells, affecting nutrient absorption and thus inhibiting normal root growth. Salt is also capable of causing ionic toxicity in plants, leading to an ionic imbalance in the plants, affecting the osmotic pressure balance inside and outside the root cells, and leading to cell swelling or shrinkage and, in severe cases, cell death. Salt, therefore, plays an important role in plant growth and development [81,82,83]. Huang et al. isolated a WRKY family transcription factor, OsWRKY50, and *OsWRKY50* overexpression lines enhanced salt stress tolerance in plants. *OsWRKY50* transcription was repressed under salt stress conditions but was activated after ABA treatment. OsWRKY50 was able to bind to the promoter of *OsNCED5* and repress its transcription [84]. Huang et al. identified a new WRKY family transcription factor, OsWRKY54. Salt stress resulted in a rapid induction in *OsWRKY54* expression in roots. The *wrky54* mutant led to greater sodium accumulation in shoots and enhanced the sensitivity of rice plants to salt stress. OsWRKY54 regulates the expression of *OsHKT1;5*; this is an essential gene related to salt tolerance. OsWRKY54 regulates the expression of *OsHKT1;5* by directly binding to the W-box motif in its promoter [85]. Fang et al. found a new transcription factor gene, *ZmWRKY86,* in *Zea mays* L., whose expression was upregulated by salt stress. The *wrky86* mutant enhanced plants’ tolerance to salt stress, with higher viability, catalase activity, and K^+^ contents, and lower malondialdehyde accumulation and Na^+^ contents under salt stress conditions [86]. Yu et al. found that *TaWRKY17* expression was upregulated by salt, drought, hydrogen peroxide (H_2_O_2_), and ABA treatments in wheat. Overexpression of *TaWRKY17* in *Arabidopsis thaliana* and wheat resulted in a significant increase in the plants’ salt stress tolerance. Among the *TaWRKY17* overexpression plants, SOD, POD, and CAT activities were elevated, whereas H_2_O_2_ and MDA accumulation were reduced [87]. 

### 2.4. Role of WRKY Transcription Factors in Plants’ Response to Heavy Metal Stress

Heavy metal levels in plants that exceed thresholds can affect produce quality and food safety. Heavy metals can also inhibit the growth of plants, resulting in short plants, the loss of green leaves, poor root development, and other phenomena. Heavy metals can interfere with the normal physiological and metabolic processes of plants, affecting photosynthesis and respiration, leading to poor nutrient uptake and ultimately causing the death of plants and affecting plant yields. If heavy metals excessively accumulate in food crops, human consumption of such crops will cause great harm to human health. Therefore, controlling heavy metal levels is essential for human food security [88,89,90,91,92]. Gu et al. identified that the WRKY transcription factor gene *ZmWRKY64* is enhanced in maize roots and leaves under cadmium stress and that knocking down the expression of *ZmWRKY64* leads to excessive cadmium accumulation in leaf and root cells, resulting in a cadmium-sensitive phenotype. *ZmSRG7* is a key gene that regulates ROS homeostasis under abiotic stress, and ZmWRKY64 directly enhances the transcription of this gene, thereby regulating maize tolerance in response to cadmium stress [93]. Jia et al. identified a WRKY transcription factor, TaWRKY70, that regulates the tolerance to the heavy metal cadmium in wheat. Cadmium accumulated in *TaWRKY70-*overexpressing *Arabidopsis* roots but not in leaf tissues. When *TaWRKY70* was expressed, the net influx of Cd^2+^ into *Arabidopsis* roots was reduced. The overexpression of *TaWRKY70* in *Arabidopsis thaliana* showed lower electrolyte leakage and malondialdehyde and hydrogen peroxide contents than in the wild type and higher antioxidant enzyme activities than in the wild type. TaWRKY70 directly binds to and regulates the expression of the *TaCAT5* promoter, which, in turn, regulates the tolerance of plants to cadmium stress [94]. Xian et al. identified a WRKY transcription factor gene, *GmWRKY172*, whose expression was significantly upregulated under cadmium stress. *GmWRKY172* overexpression lines exhibited an enhanced cadmium tolerance and reduced cadmium content in shoots. Under cadmium stress, transgenic soybean accumulated less MDA and H_2_O_2_ and had higher flavonoid contents, lignin contents, and POD activity than the wild type [95]. Under cadmium stress, the *StWRKY6* overexpression strain had significantly higher soil and plant analysis development values and reactive oxygen-scavenging enzyme contents than the wild type. The ability of cadmium to induce StWRKY6 transcription factors upregulated the expression of a number of potential genes, including those involved in cadmium chelation, such as *APR2* and *DFRA*; plant defences, such as *VSP2* and *PDF1.4*; toxic substance efflux, such as *ABCG1*; light morphological development, such as *BBX20;* and auxin signalling, such as *SAUR64/67*. He et al. demonstrated that these genes coordinate the regulation of cadmium tolerance in *StWRKY6* overexpression lines [96]. A new WRKY transcription factor, GmWRKY142, positively regulating cadmium stress, was identified by Cai et al. *GmWRKY142* was highly expressed in roots, and the expression of this gene was significantly upregulated under cadmium stress, and the overexpression of *GmWRKY142* in Arabidopsis and soybean hairy roots significantly enhanced cadmium tolerance. *ATCDT1*, *GmCDT1-1*, and *GmCDT1-2*, encoding cadmium tolerance 1, were induced in the *GmWRKY142* overexpression lines [97].

### 2.5. WRKY Transcription Factors Involved in Plant Response to Nutritional Element Stress

Nitrogen (N) is one of the most important nutrients in the growth and development of plants and is a component of organic compounds such as proteins, chlorophyll, nucleic acids, and various biological enzymes. Plants mainly obtain inorganic nitrogen nutrients in the form of nitrate nitrogen (NO_3_^−^) and ammonium nitrogen (NH_4_^+^) in the soil through the root system. The inorganic nitrogen in the soil that can be directly used by plants is very little, and inorganic nitrogen is easy to leach and volatilise but also be fixed by the organic matter in the soil, so the effective nitrogen in the soil is far from enough for the normal growth of plants [98,99,100]. Javed et al. found four genes, *ShWRKY13-2, ShWRKY*39-1, *ShWRKY*49-3 and *ShWRKY*125-3, that exhibited significant upregulation in resistance to leaf scald LCP85-384 in two *Saccharum* spp. varieties triggered by Xanthomonas albilineans (Xa). In particular, *ShWRKY22-1*, *ShWRKY49-3*, and *ShWRKY52-1* acted as negative regulators in both *Saccharum* spp. varieties in response to a range of N injection doses [101]. To assess whether the nitrogen form affects the synthesis of the high-value terpene metabolite steviol glycosides (SGs) in stevia (*Stevia rebaudiana*), Sun et al. utilised stevia plants at the same nitrogen level with NO_3_^−^ or NH_4_^+^, and they found that the nitrogen form had no significant effect on the stevia leaf biomass or total nitrogen content, but NO_3_^−^ increased the leaf SG content. Combined transcriptome analysis identified 397 genes that were differentially expressed (DEGs) between the NO_3_^−^ and NH_4_^+^ treatments. It was concluded that NO_3_^−^ could promote leaf SG synthesis through the NO_3_^−^-MYB/WRKY-GGPPS/CPS module [102]. Betalain is a water-soluble nitrogenous pigment. Zhang et al. identified a novel WRKY transcription factor, HmoWRKY40, in *Hylocereus monacanthus*. The betalain content and *HmoWRKY40* expression rapidly increased during dragon fruit colouring, and the silencing of the *HmoWRKY40* gene led to significant reductions in the betacyanin content. HmoWRKY40 binds to the promoter of *HmoCYP76AD1* and activates its expression, thereby regulating betalain biosynthesis in *Hylocereus monacanthus* fruit [103].

Phosphorus (P) is one of the essential nutrients for plant growth and development, and phosphorus deficiency leads to morphological and physiological changes in plants. Phosphorus deficiency has an effect on photosynthesis, respiration, and biosynthetic processes in plants. Phosphorus is also an integral part of plant cells and is closely linked to all plant life activities, playing a vital role in plant growth and development [104,105,106,107]. Wang et al. identified a WRKY family of transcription factors, including OsWRKY108 and OsWRKY21, in rice, and the overexpression of these two genes resulted in the upregulation of P_i_ transporter protein genes, thereby enhancing P_i_ accumulation [108]. Wang et al. identified the FtWRKY29 transcription factor in *Fagopyrum tataricum Gaertn*. FtWRKY29 regulates the ability to tolerate phosphorus deficiency. Overexpression of *FtWRKY29* in *Arabidopsis* produced transgenic lines that increased phosphorus uptake, regulated anthocyanin accumulation, and were less sensitive to low-phosphorus-induced stress. The low-phosphorus-responsive genes *PHT1;1*, *PHT1;4*, and *PHO1* were significantly upregulated in these lines [109]. Liu et al. found that the *GmWRKY46* gene is involved in the regulation of phosphorus deficiency tolerance in soybean. The expression of *GmWRKY46* was significantly higher in low-phosphorus-sensitive soybean varieties than in phosphorus-tolerant soybean varieties, and the gene was strongly induced by phosphorus deficiency. The expression patterns of many P-responsive genes, for example, *GmPht1;1*, *GmPht1;4*, *GmPTF1*, *GmACP1*, *GmPAP21*, and *GmExpansin-A7,* were altered in the *GmWRKY46* overexpression lines and *GmWRKY46*-silenced lines [47]. Zhang et al. found that the overexpression of *OsWRKY21* or *OsWRKY108* caused an increase in P_i_ accumulation due to the elevated expression of phytophosphate transporter protein 1 (*PHT1*). *Oswrky21* and *Oswrky108* double mutants showed decreased P_i_ accumulation and *OsPHT1;1* expression in a P_i_-dependent manner. Their results demonstrate that rice WRKY transcription factors function redundantly to promote P_i_ uptake by activating *OsPHT1;1* expression under P_i_ sufficiency conditions [110]. Knockdown of *OsWRKY10* results in increased P_i_ uptake and accumulation under conditions of P_i_ sufficiency. OsPHT1;2 results in increased P_i_ accumulation in *oswrky10*. OsWRKY10 is a transcriptional repressor, induced by Pi transcription, and it is upregulated by a subset of the PHT1 gene upon its mutation. Under P_i_ starvation, the OsWRKY10 protein is degraded via the 26S proteasome. These results demonstrate that the OsWRKY10-OsPHT1;2 module inhibits P_i_ uptake only in the presence of sufficient P_i_ [111].

### 2.6. WRKY Transcription Factors and Oxidative Stress

Oxidative stress is one of the most severe stresses caused by various other stresses. There are four main types of ROS in plants: singlet oxygen (^1^O_2_), superoxide (O_2_^−^), hydroxyl radical (OH), and hydrogen peroxide (H_2_O_2_). The induction of ROS accumulation in *Arabidopsis* with salt stress or by treating plants with H_2_O_2_ or methyl viologen (MV) induces the expression of multiple genes encoding WRKY family transcription factors [112,113,114,115]. Jia et al. identified a WRKY family of transcription factors, GhWRKY68. Promoter-driven β-glucuronidase activity is enhanced after exposure to drought, salt, ABA, and H_2_O_2_. *GhWRKY68* overexpression lines showed reduced resistance to drought and salt and reduced tolerance to oxidative stress, which was associated with the accumulation of ROS, reduced enzyme activity, increased MDA contents, and an altered expression of ROS-related genes [116]. Sun et al. found that *AtWRKY53* overexpression lines were highly sensitive to drought stress compared with Col-0 plants. The activated expression of *AtWRKY53* inhibited stomatal closure by reducing the H_2_O_2_ content in guard cells. AtWRKY53 could bind directly to the qua-quine starch (*QQS*, AT3G30720) gene promoter sequence, leading to enhanced starch metabolism [117].

## 3. Conclusions and Prospects

Due to the continuous changes in the global climatic environment, plants often suffer from different forms of biotic and abiotic stress during their growth and development. In order to cope with these adverse conditions, plants have developed a complete coping system in the process of evolution. Plant adversities are dealt with through the protein regulation of the expression levels of downstream genes or interactions between proteins. Because transcription factors contain different gene-binding sites, they can often regulate the expression of multiple downstream genes. Therefore, focusing on the functions of transcription factors has become a major issue in crops. As a large class of important transcription factors in plants, WRKY transcription factor genes play a key role throughout a plant’s life cycle. With the continuous development of gene editing and third-generation sequencing technology, many researchers have verified the functions of WRKY family members in different types of plants and have found and proved that WRKY genes play a key role in plant growth and development and biotic and abiotic stresses [118,119,120,121,122]. In order to investigate the phylogeny and evolutionary relationships between WRKY family genes in different species, we constructed phylogenetic trees of the amino acid sequences of some WRKY family transcription factors in *Glycine max*, *Arabidopsis thaliana Oryza sativa*, and other plants using the MEGA 11.0 software [123]. Among them, we found that OsWRKY97 and SgWRKY11 were highly related; HvWRKY72 and TaWRKY72 were highly related; SbWRKY72 and ZmWRKY61 were highly related; OsWRKY90 and PhWRKY63 were highly related; GmWRKY58 and GmWRKY76 were highly related; PbWRKY45 and PdWRKY22-like were highly related; ZmWRKY22, SbWRKY22, PaWRKY22-like, DoWRKY22, and SiWRKY22 were highly related; and OsWRKY87 and AtWRKY39 were highly related, which indicated that the WRKY family of transcription factors are highly related in various species and have close affinities between them, and it is possible that WRKY family transcription factors have similar functions in different species (Figure S1). 

Future research on WRKY transcription factors can start from the following aspects, such as the use of CRISPR/Cas9 technology to knock out the WRKY family transcription factor genes and the overexpression of genes and other technologies to cultivate new varieties of resilient and excellent crops and to promote the sustainable development of agriculture; to further investigate the upstream and downstream regulators and target genes of WRKY family transcription factor genes by using existing technologies; to analyse their regulatory networks in response to adversity stress; and to elucidate the molecular mechanisms of WRKY and plants’ responses to abiotic stresses.

Nowadays, scientists mainly focus on the response of WRKY transcription factors to common abiotic stresses such as drought, cold, salt, and heavy metals (Table 2). Meanwhile, the molecular mechanisms of WRKY transcription factors in response to chemical agent stresses are less reported. In today’s increasingly developed industry, chemical pollutants such as car exhaust, haze, and pesticides are extremely harmful to crops. The main component of automobile exhaust and haze is sulphide, and SO_2_ in the atmosphere generates SO_3_ under the action of sunlight, water vapour, drifting dust, and so on. Firstly, SO_3_ falls to the ground in the form of rainfall and drenches plants, damaging the waxy protective layer of the epidermis of the plant leaves and impairing the normal transpiration and gas exchange process. Secondly, acid rain also destroys a class of alkaline nutrients such as potassium, calcium, and phosphorus in the soil, leading to the withering and death of plants that cannot absorb nutrients in soils with insufficient fertility. The excessive use of pesticides inhibits the growth and development of plants, resulting in short plants, a small leaf area, and the yellowing of the leaf colour. The toxicity of pesticides can also lead to cell membrane rupture and cytoplasmic leakage and ultimately affects the normal growth and development of plants. Today, people are more and more concerned about crop security. If we pay more attention to the molecular mechanisms of WRKY transcription factors and chemical reagents in the future, this will provide a guarantee for crop security.

In summary, the WRKY family of transcription factors is critical for plant growth and development as well as regulation in plants’ response to abiotic stresses. In the future, we will pay more attention to the mechanisms by which WRKY transcription factors can regulate downstream target genes in response to abiotic stresses, which can provide a more theoretical basis for the improvement of food safety.

## Figures and Tables

**Table 1:** The *WRKY* genes’ total numbers in different plants.

Gene Name	Species	Total Number	Reference
*AtWRKYs*	*Arabidopsis thaliana*	74	[29]
*OsWRKYs*	*Oryza sativa*	100+	[30]
*GmWRKYs*	*Glycine max*	197	[31]
*HvWRKYs*	*Hordeum vulgare*	45	[32]
*CsWRKYs*	*Cucumis sativus*	55	[33]
*SlWRKYs*	*Solanum lycopersicum*	81	[34]
*PgWRKYs*	*Panax ginseng*	118	[35]
*VuWRKYs*	*Vigna unguiculata*	92	[36]
*HvWRKYs*	*Hordeum vulgare*	86	[37]
*IbWRKYs*	*Ipomoea batatas*	84	[38]
*PhWRKYs*	*Petunia hybrida*	79	[39]
*TkWRKYs*	*Taraxacum kok-saghyz*	72	[40]
*SbWRKYs*	*Scutellaria baicalensis*	72	[41]
*HuWRKYs*	*Hylocereus undulatus*	70	[42]
*DcWRKYs*	*Daucus carota*	67	[43]
*XsWRKYs*	*Xanthoceras sorbifolium*	65	[44]
*KoWRKYs*	*Kandelia obovata*	64	[45]

**Table 2:** Abiotic stress-responsive WRKY transcription factors in plants.

Abiotic Stress Type	WRKY Transcription Factors	Species	Expression Pattern	Reference
Heat	AtWRKY39	*Arabidopsis thaliana* L.	Increased	[124]
Boron	AtWRKY47	*Arabidopsis thaliana* L.	Decreased	[125]
Cadmium	AtWRKY13	*Arabidopsis thaliana* L.	Increased	[126]
Salt	AtWRKY28	*Arabidopsis thaliana* L.	Increased	[127]
Salt	AtWRKY33	*Arabidopsis thaliana* L.	Increased	[128]
Salt	AtWRKY46	*Arabidopsis thaliana* L.	Increased	[129]
Salt and drought	GmWRKY12	*Glycine max* L.	Increased	[130]
Salt and drought	GmWRKY16	*Glycine max* L.	Increased	[131]
Salt	GmWRKY20	*Glycine max* L.	Increased	[132]
Salt and drought	GmWRKY27	*Glycine max* L.	Increased	[133]
Salt and drought	GmWRKY54	*Glycine max* L.	Increased	[134]
Salt	GmWRKY144, 165	*Glycine max* L.	Increased	[135]
Salt	ZmWRKY17	*Zea mays* L.	Decreased	[136]
Salt	ZmWRKY86	*Zea mays* L.	Decreased	[86]
Drought	ZmWRKY104	*Zea mays* L.	Increased	[137]
Salt	OsWRKY50	*Oryza sativa*	Increased	[84]
Cold	OsWRKY63	*Oryza sativa*	Decreased	[18]
Cold	OsWRKY76	*Oryza sativa*	Increased	[138]
Salt	OsWRKY42	*Oryza sativa*	Increased	[139]
Drought	OsWRKY55	*Oryza sativa*	Decreased	[140]
Drought	OsWRKY5	*Oryza sativa*	Decreased	[141]
Drought	OsWRKY97	*Oryza sativa*	Increased	[142]
Aluminium	OsWRKY22	*Oryza sativa*	Decreased	[143]
Salt and drought	OsWRKY87	*Oryza sativa*	Increased	[144]

## Data Availability

Not applicable.

## Supplementary Materials

The following supporting information can be downloaded at https://www.mdpi.com/article/10.3390/ijms25136845/s1.
