# Supplementary material for: WRKY Transcription Factor Responses and Tolerance to Abiotic Stresses in Plants
Source: Int J Mol Sci. 2024 Jun 21;25(13):6845. doi: 10.3390/ijms25136845 (PMC11241455; doi:10.3390/ijms25136845)
Supplement: Supplementary file 1 [file ijms-25-06845-s001.zip › ijms-3064354-supplementary/ijms-3064354-figures.pdf]

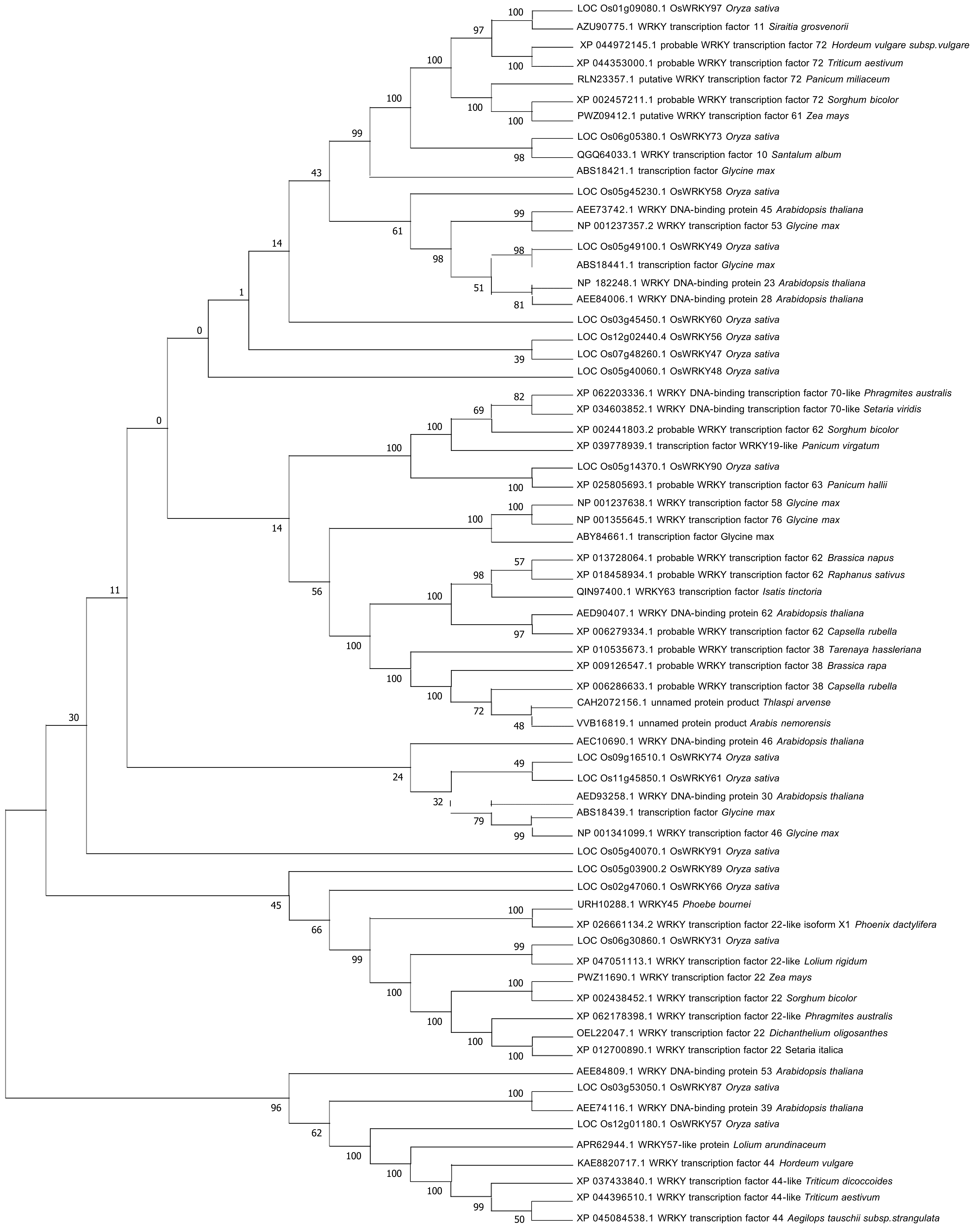
LOC Os01g09080.1 OsWRKY97 *Oryza sativa*
AZU90775.1 WRKY transcription factor 11 *Siraitia grosvenorii*
XP 044972145.1 probable WRKY transcription factor 72 *Hordeum vulgare subsp.vulgare*
XP 044353000.1 probable WRKY transcription factor 72 *Triticum aestivum*
RLN23357.1 putative WRKY transcription factor 72 *Panicum miliaceum*
XP 002457211.1 probable WRKY transcription factor 72 *Sorghum bicolor*
PWZ09412.1 putative WRKY transcription factor 61 *Zea mays*
LOC Os06g05380.1 OsWRKY73 *Oryza sativa*
QGQ64033.1 WRKY transcription factor 10 *Santalum album*
ABS18421.1 transcription factor *Glycine max*
LOC Os05g45230.1 OsWRKY58 *Oryza sativa*
AEE73742.1 WRKY DNA-binding protein 45 *Arabidopsis thaliana*
NP 001237357.2 WRKY transcription factor 53 *Glycine max*
LOC Os05g49100.1 OsWRKY49 *Oryza sativa*
ABS18441.1 transcription factor *Glycine max*
NP 182248.1 WRKY DNA-binding protein 23 *Arabidopsis thaliana*
AEE84006.1 WRKY DNA-binding protein 28 *Arabidopsis thaliana*
LOC Os03g45450.1 OsWRKY60 *Oryza sativa*
LOC Os12g02440.4 OsWRKY56 *Oryza sativa*
LOC Os07g48260.1 OsWRKY47 *Oryza sativa*
LOC Os05g40060.1 OsWRKY48 *Oryza sativa*
XP 062203336.1 WRKY DNA-binding transcription factor 70-like *Phragmites australis*
XP 034603852.1 WRKY DNA-binding transcription factor 70-like *Setaria viridis*
XP 002441803.2 probable WRKY transcription factor 62 *Sorghum bicolor*
XP 039778939.1 transcription factor WRKY19-like *Panicum virgatum*
LOC Os05g14370.1 OsWRKY90 *Oryza sativa*
XP 025805693.1 probable WRKY transcription factor 63 *Panicum hallii*
NP 001237638.1 WRKY transcription factor 58 *Glycine max*
NP 001355645.1 WRKY transcription factor 76 *Glycine max*
ABY84661.1 transcription factor Glycine max
XP 013728064.1 probable WRKY transcription factor 62 *Brassica napus*
XP 018458934.1 probable WRKY transcription factor 62 *Raphanus sativus*
QIN97400.1 WRKY63 transcription factor *Isatis tinctoria*
AED90407.1 WRKY DNA-binding protein 62 *Arabidopsis thaliana*
XP 006279334.1 probable WRKY transcription factor 62 *Capsella rubella*
XP 010535673.1 probable WRKY transcription factor 38 *Tarenaya hassleriana*
XP 009126547.1 probable WRKY transcription factor 38 *Brassica rapa*
XP 006286633.1 probable WRKY transcription factor 38 *Capsella rubella*
CAH2072156.1 unnamed protein product *Thlaspi arvense*
VVB16819.1 unnamed protein product *Arabis nemorensis*
AEC10690.1 WRKY DNA-binding protein 46 *Arabidopsis thaliana*
LOC Os09g16510.1 OsWRKY74 *Oryza sativa*
LOC Os11g45850.1 OsWRKY61 *Oryza sativa*
AED93258.1 WRKY DNA-binding protein 30 *Arabidopsis thaliana*
ABS18439.1 transcription factor *Glycine max*
NP 001341099.1 WRKY transcription factor 46 *Glycine max*
LOC Os05g40070.1 OsWRKY91 *Oryza sativa*
LOC Os05g03900.2 OsWRKY89 *Oryza sativa*
LOC Os02g47060.1 OsWRKY66 *Oryza sativa*
URH10288.1 WRKY45 *Phoebe bournei*
XP 026661134.2 WRKY transcription factor 22-like isoform X1 *Phoenix dactylifera*
LOC Os06g30860.1 OsWRKY31 *Oryza sativa*
XP 047051113.1 WRKY transcription factor 22-like *Lolium rigidum*
PWZ11690.1 WRKY transcription factor 22 *Zea mays*
XP 002438452.1 WRKY transcription factor 22 *Sorghum bicolor*
XP 062178398.1 WRKY transcription factor 22-like *Phragmites australis*
OEL22047.1 WRKY transcription factor 22 *Dichanthelium oligosanthes*
XP 012700890.1 WRKY transcription factor 22 Setaria italica
AEE84809.1 WRKY DNA-binding protein 53 *Arabidopsis thaliana*
LOC Os03g53050.1 OsWRKY87 *Oryza sativa*
AEE74116.1 WRKY DNA-binding protein 39 *Arabidopsis thaliana*
LOC Os12g01180.1 OsWRKY57 *Oryza sativa*
APR62944.1 WRKY57-like protein *Lolium arundinaceum*
KAE8820717.1 WRKY transcription factor 44 *Hordeum vulgare*
XP 037433840.1 WRKY transcription factor 44-like *Triticum dicoccoides*
XP 044396510.1 WRKY transcription factor 44-like *Triticum aestivum*
XP 045084538.1 WRKY transcription factor 44 *Aegilops tauschii subsp.strangulata*

Figure S1. Phylogenetic tree WRKYs family genes in *Glycine max*, *Arabidopsis thaliana*, *Oryza sativa* and so on. Phylogenetic tree of WRKY transcription factors proteins in selected angiosperms. The WRKY family gene sequences of plants were retrieved using the China National Rice Data Center (https://www.ricedata.cn/) and NCBI (https://www.ncbi.nlm.nih.gov/). The phylogenetic tree was constructed using MEGA version 11.0 with the bootstrap method based on full amino acid sequences. Numbers next to the branches show the percentage of replicate trees in which the associated taxa clustered together in the bootstrap test (1000 replicates).
